# DOES MUSICKING IMPROVE WELL-BEING IN LATER LIFE? USING BASIC PSYCHOLOGICAL NEEDS THEORY TO EXPLORE THE QUESTION

**DOI:** 10.1093/geroni/igz038.1144

**Published:** 2019-11-08

**Authors:** Anna Wong, Koji Matsunobu

**Affiliations:** 1 The Chinese University of Hong Kong, Shatin, New Territories, Hong Kong; 2 The Education University of Hong Kong, Tai Po, New Territories, Hong Kong

## Abstract

Older adults are vulnerable to developing poor mental health as they experience significant life changes such as retirement, widowhood, living alone, institutionalisation, physical and/or cognitive deterioration in oneself or one’s partner, which are associated with increased depression and loneliness. Understanding the mechanisms and identifying effective measures that strengthen their capacities to cope are therefore very important. Extensive research has suggested that active music-making with others has many psychosocial benefits for older adults. This study explored in detail the musical experiences drawn from different settings of Japanese and Hong Kong music communities. Semi-structured group interviews were conducted for members of music-making groups in Japan and Hong Kong who regularly practised, rehearsed, and performed their instruments together in community settings. A phenomenological approach was used to capture and analyse their lived experiences. A needs satisfaction theoretical framework was adopted to shed light on links between their musical engagement and wellbeing outcomes. Active musical engagement was found to be an important source of support for older musicians’ psychological needs. It was central to their positive identity development and sense of purpose in old age. The presentation will further elaborate on underlying mechanisms that linked social, emotional, and artistic experiences of active musical engagement to wellbeing. This study identified active musical engagement as an effective agent in healthy ageing. Differential manifestations of need-supportive practices in musically and culturally distinct communities in Japan and Hong Kong were also described, giving evidence for the positive value of community music groups for promoting mental health and wellbeing.

